# Tumor Cell Death Mediated by Peptides That Recognize Branched Intermediates of DNA Replication and Repair

**DOI:** 10.1371/journal.pone.0078751

**Published:** 2013-11-14

**Authors:** Mamon Dey, Sukanya Patra, Leo Y. Su, Anca M. Segall

**Affiliations:** Department of Biology and Center for Microbial Sciences, San Diego State University, San Diego, California, United States of America; National Cancer Institute, United States of America

## Abstract

Effective treatments for cancer are still needed, both for cancers that do not respond well to current therapeutics and for cancers that become resistant to available treatments. Herein we investigated the effect of a structure-selective d-amino acid peptide wrwycr that binds replication fork mimics and Holliday Junction (HJs) intermediates of homologous recombination (HR) *in vitro*, and inhibits their resolution by HJ-processing enzymes. We predicted that treating cells with HJ-binding compounds would lead to accumulation of DNA damage. As cells repair endogenous or exogenous DNA damage, collapsed replication forks and HJ intermediates will accumulate and serve as targets for the HJ-binding peptides. Inhibiting junction resolution will lead to further accumulation of DNA breaks, eventually resulting in amplification of the damage and causing cell death. Both peptide wrwycr and the related wrwyrggrywrw entered cancer cells and reduced cell survival in a dose- and time-dependent manner. Early markers for DNA damage, γH2AX foci and 53BP1 foci, increased with dose and/or time exposure to the peptides. DNA breaks persisted at least 48 h, and both checkpoint proteins Chk1 and Chk2 were activated. The passage of the cells from S to G2/M was blocked even after 72 h. Apoptosis, however, was not induced in either HeLa or PC3 cells. Based on colony-forming assays, about 35% peptide-induced cytotoxicity was irreversible. Finally, sublethal doses of peptide wrwycr (50–100 µM) in conjunction with sublethal doses of several DNA damaging agents (etoposide, doxorubicin, and HU) reduced cell survival at least additively and sometimes synergistically. Taken together, the results suggest that the peptides merit further investigation as proof-of-principle molecules for a new class of anti-cancer therapeutics, in particular in combination with other DNA damaging therapies.

## Introduction

Cancer is a consortium of life threatening diseases with no definitive treatment. Although therapies specifically targeted to individual cancer types may be the ideal treatment, this goal remains distant and general therapies are still useful. Tumor cells have faster replication rates and higher metabolism, and are likely subjected to more DNA damage than normal cells. Many tumor cells bear mutations that reduce the efficacy of repair mechanisms and the responses to accumulating damage. At the same time, many of the effective anti-cancer therapies currently in use, including radiation, are DNA damaging agents [1]–[4] that generate double strand DNA breaks (DSBs). The resulting DNA damage overwhelms the DNA repair capacity of the tumor cells, leading to cell cycle arrest and cell death. Currently, the option of using DNA damaging agents that induce specific damage in conjunction with different DNA repair inhibitors is being explored to improve the cancer treatment [5]–[8].

Eukaryotic cells repair DSBs using homologous recombination repair (HRR) [9], [10] and non-homologous end joining (NHEJ) [11]–[13]. Homologous recombination (HR) is the major error-free double strand DNA repair pathway and usually occurs in late S-phase of the cell cycle when sister chromatids are available as templates for repair. During HR-dependent repair, transient DNA intermediates known as Holliday or 4-way junctions (HJ) are generated and must be resolved to permit correct segregation of chromatids at mitosis [14]–[17]. Many cancer cells are predicted to produce more HJ intermediates than most non-neoplastic cells.

Our laboratory previously identified peptides and small molecules that bind to Holliday junctions and replication fork mimics, branched DNA structures that are intermediates in DNA repair [18]–[23]. Among the most potent such molecules was the d-amino acid hexapeptide wrwycr (Trp-Arg-Trp-Tyr-Cys-Arg), whose active form is a disulfide-bridged homodimer. In addition to binding HJs *in vitro*, peptide wrwycr also binds Holliday junctions *in vivo* [24] and is synergistically toxic with DNA-damaging agents including mitomycin C, UV, H_2_O_2_, and topoisomerase inhibitors in bacteria ([25]; Naili, Rostron and Segall, ms. submitted). The peptide has no discernible DNA sequence specificity; rather it is structure-selective, with the higher affinity for HJs than replication forks by 2–5 fold [20], [22], [26].

The objective of this study was to investigate the effects of peptide wrwycr in cancer cells and to explore the mechanism of action of the peptide. We demonstrated that wrwycr enters the cells in a dose dependent manner and decreases cellular viability in a manner independent of apoptosis. We hypothesized that the peptide binds and stabilizes HJ and/or repair-associated replication intermediates, leading to accumulation of DSBs and thereby arresting the cell cycle. Peptide treatment on its own caused the accumulation of DSBs that persisted even after 72 h, activating cell cycle checkpoints and interfering with cellular progression beyond S-phase. We further tested whether, as predicted by the hypothesis, sublethal doses of the peptide are synergistic with sublethal doses of DNA damaging chemotherapeutics such as etoposide and doxorubicin.

As mentioned earlier, the active form of wrwycr is a disulfide-bridged dimer. Because conditions within cells may reduce dimers into monomers, we also performed some experiments with peptide wrwyrggrywrw (aka dodecamer). This peptide was synthesized as an inverted repeat of the d-wrwycr dimer but replacing the cysteines with glycines and moving their position within the peptide by one amino acid. Data obtained previously indicated that this single chain dodecapeptide is a good mimic of the dimerized form of wrwycr, and is active at lower concentrations, presumably because it is not dependent on the disulfide bridge [27]. Indeed, the effects of this dodecapeptide mimic well those of wrwycr, and at lower peptide concentrations. However, this peptide has higher nonspecific DNA binding activity and toxicity, and for this reason we have not adopted it as our model compound.

## Materials and Methods

### Cell culture and peptides

No animal or human subjects were used in this work. The cancer cell lines used for the study were A549, PC3, DU145, LnCAP, DuPro-1, PPC-1 and HeLa. PC3, PPC-1 and DuPro-1 were generous gifts of Drs Doug Burton and Leonard Deftos in VA Medical Center, UCSD; A549, Du145 and LnCAP were generous gifts from Dr. Gerrard Boss in the Department of Medicine, USCD Medical School). The original source of all the cell lines except for DuPro-1 is ATCC. DuPro-1 was originally isolated from a xenograft model, by William Isaacs (Johns Hopkins Univ., Baltimore, MA) [28], [29]. They were grown in 75 cm^2^ bottle canted necked vented flasks (Corning) with DMEM (Mediatech Inc, Manassas, VA), except for PC3 which was grown in RPMI with L-glutamine (Mediatech Inc, Manassas, VA), supplemented with 10% FBS at 37°C in a humidified incubator with 5% CO_2_–95% O_2_ flow.

All the peptides used in this study were synthesized from D amino acids and with an amide group at the C terminus either by Sigma Genosys or Biosynthesis Inc. The purity of the peptides was >95% as gauged by HPLC and verified by mass spectrometry, and were maintained as 10 mM stocks in 100% DMSO at −20°C. The dimerization extent of the peptide wrwycr was monitored by HPLC, and the working stocks used contained ∼80% dimerized wrwycr (also known as d8). The Cys5Ala substitution in peptide d-wrwyar prevents disulfide-based dimerization and renders this peptide less than 50× as active as d-wrwycr, helping us probe the specificity of the activities of peptide wrwycr [19]. Peptide d-wkhyny, with similar properties to d-wrwyar (but more extensively characterized) was similarly used as a “negative” control [15], [16], [17], [19].

### Quantification of cell viability by MTT assay

20,000 PC3 cells were plated overnight in 96 well plates (Costar) at a concentration of 200,000 cells/ml. They are treated with the peptides, doxorubicin, docetaxel alone, or in combination for different time period incubation and the cell viability was determined by MTT assay as described [30]. The percentage survival of the cells with different treatments was calculated using DMSO-vehicle control-treated cells as the standard.

### Live/Dead assay

PC3 or HeLa cells were seeded at 400,000 or 500,000 cells per well respectively in 6-well tissue culture plates (BD Falcon) and allowed to attach overnight at 37°C with 5% CO_2_. The cells were incubated with different concentrations of d-wrwycr for 24 or 48 h respectively before the cells were lifted off from the plate. Cell pellets were resuspended in 200 µl of dye solution containing either cell-permeable calcein AM and/or cell-impermeant ethidium homodimer according to the manufacturer's protocol (Live/dead cytotoxicity kit for mammalian cells, Invitrogen and reference [30]). Data were collected in BDFacs Canto and analyzed using FACSDiva software (Becton-Dickenson).

### Colony forming assay

HeLa cells were seeded at 500 cells per well in 6-well tissue culture plates and allowed to attach overnight at 37°C with 5% CO_2_. The media was replaced with fresh DMEM containing the appropriate treatments and incubated for 24 h. After the treatment solutions were removed and the cells were washed once with 1× PBS, fresh peptide-free media were added and the cells were allowed to grow for 6–7 days (when individual colonies became visible under the microscope). Following removal of the media and washing once with 1× PBS, the cells were stained with 1% crystal violet solution for 2 min at room temperature then immediately rinsed under running water until all excess stain were washed from the plates. Finally, the number of colonies was counted for each treatment and % survival was calculated taking media treated colonies as 100% survival. At least three independent experiments, each done in triplicate, were performed for each treatment condition.

### High Performance Liquid Chromatography (HPLC)

500,000 HeLa or 400,000 PC3 cells were plated in each well of the 6-well dishes (Costar) and allowed to attach overnight at 37°C with 5% CO_2_. The cells were treated with d-wrwycr or d-wrwyrggrywrw for 24 or 48 h respectively. For PC3 cells media along with the dodecamer treatments were collected for a separate run in HPLC. PC3 or HeLa cells were lysed using a lysis buffer containing 0.71% NP40, 71 mM Tris (pH 7.5), 0.71 mM EDTA and 212 mM NaCl on ice for 5–10 mins. The lysates were centrifuged at 13,000 g for 7 mins to clear cellular debris. The samples were analyzed by HPLC (Beckman Coulter System Gold BioEssential 126/168) using a Phenomonex Jupiter 4 micron Proteo 90A C18 hydrophobic column to detect the peptides within the cells. The peptides were eluted with 100% acetonitrile supplemented with 0.1% TFA following a method using an acetonitrile gradient of 5–30% from 0 to 13.5 mins, 30–45% from next 13.5 to 28.5 min and a final 100% acetonitrile for the last 28.5 to 40 mins at a flow rate of 1 ml/min. The standards of the peptide from 0.25 µM through 75 µM concentration were run to obtain a standard curve. The intracellular concentration was calculated by taking into account the area under the peptide peaks, using the integration function of 32 Karat software (Beckman Coulter, Fullerton, California), and comparing them to the standard curve of the peptide. The intracellular peptide concentration per sample thus obtained was divided by the number of cells per sample (500,000 for HeLa or 400,000 for PC3 cells) and the intracellular volume of the cell (2.5 pl per cell for HeLa cells) [31], [32]. The extracellular concentration of wrwyrggrywrw was calculated by running the media similarly in HPLC and comparing them to the standard curve of wrwyrggrywrw.

### Caspase-3 assay

400,000 PC3 cells were plated in a 6-well plate (BD Falcon) at a concentration of 200,000 cells/ml and were allowed to attach overnight at 37°C with 5% CO_2_ and treated with 50–200 µM wrwycr for 12, 24 or 48 h. The wrwycr-treated cell lysates were incubated with Ac-DEVD (Asp-Glu-Val-Asp)-AFC, a caspase-3 substrate, for 1 h. Activated caspase-3 will cleave between the DEVD and AFC, releasing latter, a fluorogenic compound that can be excited at 400 nm and the resulting emission can be read at 505 nm by the Spectra Max Gemini XS fluorometer plate reader using Softmax Pro version 3.1.2 software. The total protein concentration was calculated for each sample by using BioRad protein assay kit. To normalize the fluorescence reading (RFU) for each sample, the reading for each sample was divided by the corresponding protein concentration of the samples.

### Cytochrome C release assay for detecting apoptosis

200,000 Htog1 cells (variant of HeLa cells, a generous gift from Drs. Terrence Frey and Maria Guo) were seeded on cover slips mounted on 35 mm microwell petri dishes (Mattek Corp, Ashland, MA) with DMEM supplemented with 10% FBS and allowed to attach overnight at 37°C in 5% CO_2_. The media was replaced with 1 ml of the appropriate treatment and incubated for 24 h. After 23 h of treatment, 50 µM tetramethyl rhodamine ethyl ester perchlorate (TMRE) solution was added to each well (final concentration 50 nM) and incubated for the remaining hour. Cytochrome C release and loss of TMRE staining were determined in Z-series images acquired using a Leica TCS SP2 inverted confocal microscope. GFP was excited using 488 nm wavelength from an Ar/Kr laser attenuated to 33%, and the emitted fluorescence detected at 497–553 nm. TMRE fluorescence was excited using a 543 nm wavelength from an Ar/Kr laser attenuated to 35%, and the emitted fluorescence detected at 555–620 nm. Each cell was analyzed for release of GFP (GFP channel), loss of mitochondrial membrane potential (TMRE loss) or morphological abnormalities associated with apoptosis, and the % cells positive for each hallmark was calculated for the various treatments.

### Quantification of γH2AX and 53BP1 foci by fluorescence microscopy

200,000 PC3 cells were plated on cover-slips (Fisherbrand) mounted on each well of the 12- well dishes and allowed to attach overnight at 37°C incubator with 5% CO_2_. The cells were treated with 50, 100, 150, or 200 µM of wrwycr, or 1, 5, 10, 25, or 50 µM of wrwyrggrywrw for 48 h. The cells are fixed with 4% paraformaldehyde, permeabilized with 0.1% Triton X-100 lysis buffer and subsequently blocked with 5% BSA. The samples were probed with 1∶500 diluted mouse anti-γH2AX antibody (Millipore) or 1∶1000 diluted rabbit anti-53BP1 (Novus-Biologicals) with overnight incubation followed by Dylight anti-mouse or anti-rabbit secondary antibodies (Biolegend) respectively. DAPI (5 µg/ml) was used to stain the nucleus. The stained cells on the coverslips were mounted on the glass slides and observed with a Zeiss inverted microscope with AxioVision 4.8 software and using the 100× magnification. Foci of 53BP1 were counted manually and the average number of foci per cell was calculated.

### Flow cytometry

600,000 PC3 cells were plated in 60-mm dishes (Cellstar) to allow them to attach overnight at 37°C with 5% CO_2_. They were subsequently treated with either 50–200 µM wrwycr or 50–100 µM wrwycr or 0.5–1 µM doxorubicin or combination treatment for 0, 12, 24, 36 and 48 h. The cells were fixed with 4% paraformaldehyde and permeabilized with 0.1% Triton X lysis buffer before they were probed with FITC-conjugated γH2AX antibody (Millipore), or phospho Chk2 antibody (Cell Signaling Technology) or phospho Chk1 antibody (Cell Signaling Technology) followed by probing with Dylight 488 conjugated donkey anti-rabbit secondary antibody (Biolegend) in case of pChk1 or pChk2. Samples were analyzed using a Becton Dickinson FACS Canto flow cytometer at a forward scatter voltage of 25 V, side-scatter voltage of 400 V, and FITC channel at a voltage of 400 V.

### TUNEL assay

600,000 PC3 cells or 500,000 HeLa cells were plated in 60-mm dishes or 6-well plates respectively to allow them to attach overnight at 37°C with 5% CO_2_. PC3 cells were subsequently treated with appropriate treatments for 24, 36 and 48 h while HeLa cells were treated with 1 µl of either the caspase inhibitor z-VAD-fmk or z-FA-fmk (both from Biovision), containing appropriate treatments, for 24 h. The cells were fixed with 4% paraformaldehyde for 30 min and permeabilized with 0.1% Triton X-100 in 0.1% sodium citrate buffer for 3 mins. Labeled dUTP were added along with the terminal deoxynucleotidyl transferase dUTP nick end labeling (TUNEL) (TUNEL kit from Roche) as described in the kit. Samples were analyzed using a Becton Dickinson FACS Canto flow cytometer as specified above.

### Cell cycle analysis

600,000 PC3 cells were plated in 60-mm dishes at a concentration of 200,000 cells/ml and allowed to attach before they were serum-starved for 48 h by adding RPMI with no supplemented FBS. The cells were then treated in 100, 150 or 200 µM wrwycr in RPMI supplemented with 10% FBS for either 48 h or 72 h followed by permeabilization with cold 70% ethanol overnight. RNase/PI solution (BD Pharmingen) were added to the cells following the protocol in [33] and incubated for 2.5 h at 37°C and analyzed using the PerCP-Cy5-5 channel in a FACS Canto flow cytometer. The results were analyzed using Flow-Jo software.

### Luciferase reporter assay for detecting ER stress

HeLa cells were transfected with either either 15 µg of plasmid DJT208, carrying the GRP78 promoter (pGRP78) driving the ER-stress response elements ERSE 1, ERSE 2 and ERSE 3 upstream of the luciferase gene, or 15 µg of the plasmid GL2P, carrying the luciferase gene driven by the SV40 promoter (pSV40). All cells were also co-transfected with 10 µg of the PB1 plasmid, carrying the β-galactosidase reporter transcribed by the SV40 promoter, as a transfection control. [The plasmids were generous gifts from Dr. Peter Belmont from Christopher Glembotski's Lab in SDSU]. After 24 h the cells were treated with increasing concentrations of wrwycr (100, 150 and 200 µM) for 24 h and the luciferase activity was measured using an OptocompII Luminometer. Tunicamycin (TM), a known inducer of ER stress, was used as a positive control for the experiment [34] ER stress was calculated as the fold increase of luciferase gene expression from the GRP78 promoter over that from the SV40 promoter of pGL2P for the different concentrations of the peptide over DMSO, which is the solvent for the peptide, or over the media in the case of TM. Peptide wkhyny, known to be >50 fold less effective than wrwycr in HJ accumulation [19], [35], [36], was used as a negative control for this experiment The standard error of the mean was calculated by taking into consideration results of 2 experiments performed on different days, each comprising three independent treatments.

### Statistical analysis

The statistical difference between the control and the treated samples were estimated by one-way or two-way ANOVA using Bonferroni Post-test using Graph Pad Prism Version 5 or Wilcoxon exact two-tailed test using SAS system (version 9.1; SAS, Cary, NC). The significance level was attributed at *p<0.05*.

## Results

### Peptide wrwycr decreases the cell viability in cancer cell lines

**Figure 1:**
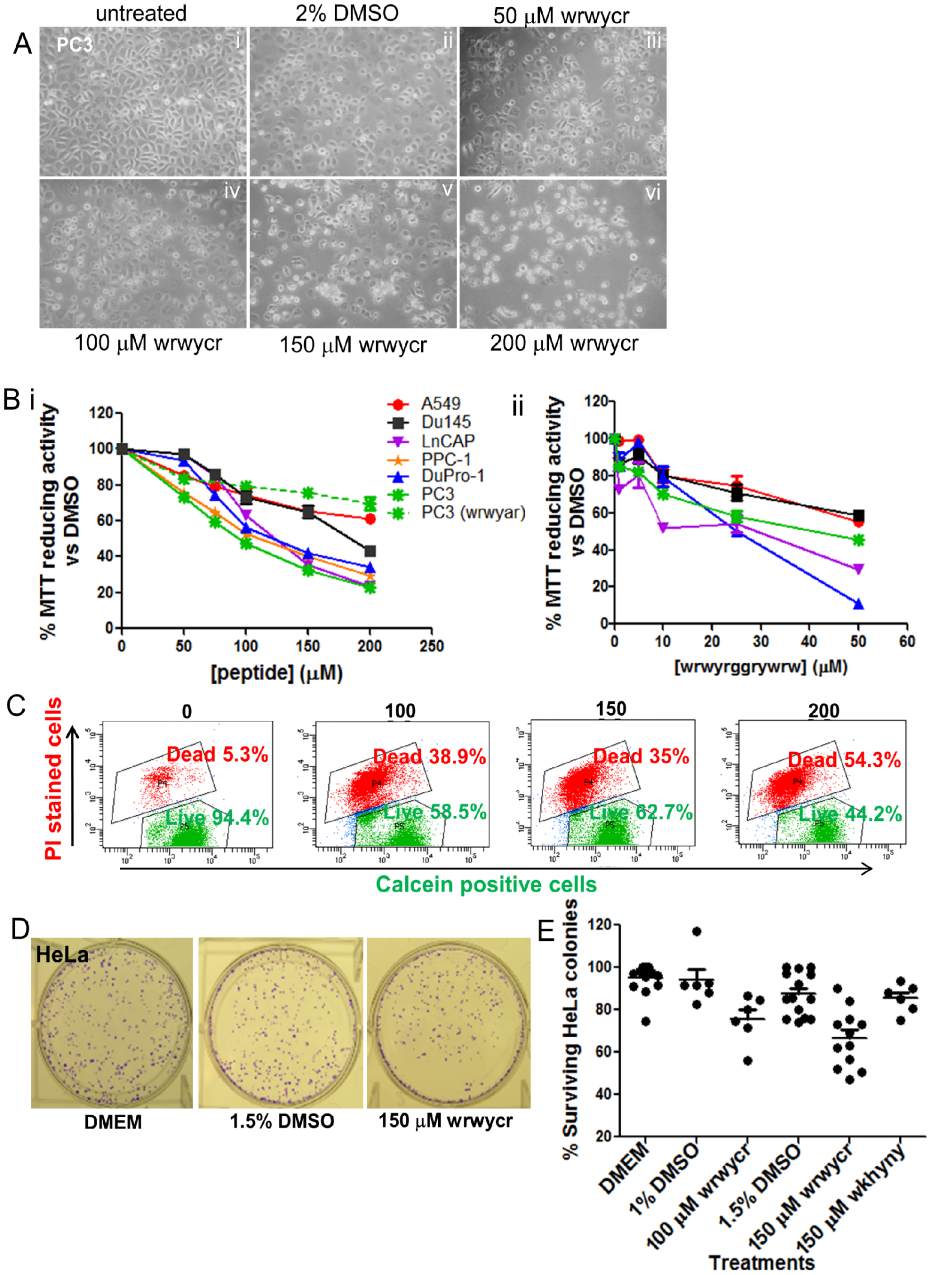
Viability assessment of cancer cell lines treated with wrwycr. **A:** Morphological changes in wrwycr-treated PC3 cells. PC3 cells were treated with 50–200 µM of wrwycr (iii–vi) for 48 h. (i): Untreated (RPMI), (ii): 2% DMSO were kept as negative controls. The cells were viewed under 25× objective using phase-contrast microscopy. The photographs were processed to greyscale using Canvas software. **B**: MTT assay using different tumor cells (PC3, Du145, LnCAP, PPC-1, DuPro-1,HeLa and A549) treated with (i): wrwycr at doses 50, 75, 100, 150, 200 µM; (ii): wrwyrggrywrw at doses 1, 5, 10, 25, or 50 µM for 48 h, except for Du145 which was treated for 24 h. MTT activity was normalized to 100% metabolic activity of cells treated with DMSO alone. **C**: Representative flow cytometry dot plots of the live-dead assay with wrwycr-treated PC3 cells using cell permeant calcein AM and cell impermeable propidium iodide dyes. Live cells are capable of converting calcein AM to fluorescent calcein (green) via nonspecific esterases. Dead cells are permeable to propidium iodide, indicating membrane disruption. **D–E**: wrwycr treatment exerts permanent damage and reduces HeLa cell viability. **D**: Representative figures of HeLa cells in colony forming assay. HeLa cells were treated for 24 h and allowed to recover and form colonies in the absence of treatment for 5–6 days. Shown here are representative colonies after treatment with (i) DMEM, (ii) 1.5% DMSO and (iii) 150 µM wrwycr. **E**: Graph summarizing the colony-forming assay results of 2 independent experiments. Reduced numbers of surviving colonies were found with 100 and 150 µM of wrwycr compared to the corresponding vehicle controls, as determined by one-way ANOVA using Bonferroni post-test analysis.

To investigate the effect of peptide wrwycr on cancer cell lines, PC3, Du145, LnCAP, DuPro-1, PPC-1, HeLa, and A549, were independently treated with different doses of wrwycr or wrwyrggrywrw for 24 or 48 h. The metabolic activity of the cell upon peptide treatment was determined by MTT and/or Live-Dead assays. Although MTT reduction is a measurement of enzymatic activity within the cells, MTT assay results are generally used as indicators of cell viability. A 50–200 µM dose of wrwycr reduced metabolic activity or survival for all cell lines in a dose dependent manner. The IC_50_ for Du145, PC3, and LnCAP cell lines was found to be 150–200 µM, 100 µM, and 100–150 µM respectively. PPC1 and DuPro1 have the same IC_50_ as the PC3 cell line (Figure 1B i). Unlike peptide wrwycr, peptide wrwyar, which does not bind HJ stably *in vitro* [16], did not reduce cell viability with increasing doses (Figure 1B i). This confirms that dimerization of peptide wrwycr is important for its cytotoxicity. To further confirm the requirement for the dimerization of wrwycr, the inverted dimer wrwyrggrywrw [37] was also used, and a similar effect on cellular viability was found. Lower doses of the single chain dodecapeptide wrwyrggrywrw (10–50 µM), compared to doses of wrwycr, were sufficient to inhibit growth of all cell lines except PPC-1 cells. After 48 h, 50 µM of wrwyrggrywrw treatment showed decrease in survival by 10%, 60%, 50% and 35% for DuPro, Du145, PC3, and LnCAP respectively. The IC_50_ of wrwyrggrywrw for DuPro and LnCAP was 25 µM and that for PC3 was 50 µM (Figure 1B ii).

The dose-dependent reduction in viability in PC3 cells correlated with the morphological changes and reduced cell number found using phase-contrast microscopy (Figure 1A). Untreated control cells (Figure 1A) were compared with the peptide-treated or DMSO-treated cells (Figure 1B). After 48 h, wrwycr-treated cells became more rounded with increasing wrwycr doses compared to the DMSO-treated cells. These morphological changes paralleled the dose effects found using the MTT assay (Figure 1B i & ii).

We tested the viability of individual PC3 and HeLa cells using the Live/Dead Viability assay and flow cytometry. Based on the results, we concluded that PC3 cells are much more sensitive to wrwycr-dependent killing than HeLa cells: PC3 cell survival decreased from 94% with DMSO treatment to 58% with 200 µM wrwycr compared to a decrease from 95% with DMSO to 79% with wrwycr in Hela cells [Table 1]. In both cases, however, the fraction of dead cells increased in a dose dependent manner, suggesting that the cytotoxicity is due to peptide wrwycr (Figure 1A). In HeLa cells, the double positive (“unhealthy”) cells were sorted and plated in the absence of peptide wrwycr, but none of these cells recovered after a week in culture [38], suggesting that wrwycr treatment of HeLa cells caused cytotoxicity but the effect was delayed compared to PC3 cells. We specifically addressed whether the cytotoxic effects of wrwycr were reversible using the colony-forming (clonogenic) assay. HeLa cells were treated for 24 h, then allowed to recover for several days in the absence of treatment. The results indicated that wrwycr caused irreversible dose dependent cytotoxicity (Figure 1D–E). The number of recovered HeLa colonies decreased from 75% to 65% in a dose dependent manner with 100–150 µM wrwycr treatment. The peptide wkhyny, which, like peptide wrwyar, is >50-fold less potent than wrwycr [19], [20], did not substantially affect the number of recovered HeLa cells (Figure 1D).

**Table 1:** Live-Dead assay on wrwycr-treated PC3 and HeLa cells using cell permeable calcein AM and cell-impermeable propidium iodide dyes.

Treatments	PC3 population		Hela population	
[wrwycr](µM)	%Live^a^	% Dead^b^	%Live	% Dead
0 (2%DMSO)	93.7±0.5	5.9±0.4	95.1±0.7	2.2±1.2
100	64.9±2.5	33.4±2.0	90.4±2.3	5.4±2.0
150	64.4±0.9	33.8±0.6	86.3±2.5	7.8±2.7
200	57.7±4.7	40.6±4.8	79.3±4.5	12.7±6.0

a Live cells are those capable of converting calcein AM to fluorescent calcein by their esterase activity.

b Dead cells are permeable to propidium iodide, indicating membrane disruption.

Standard errors are shown for at least 4 independent replicates on at least 2 separate days.

**Figure 2:**
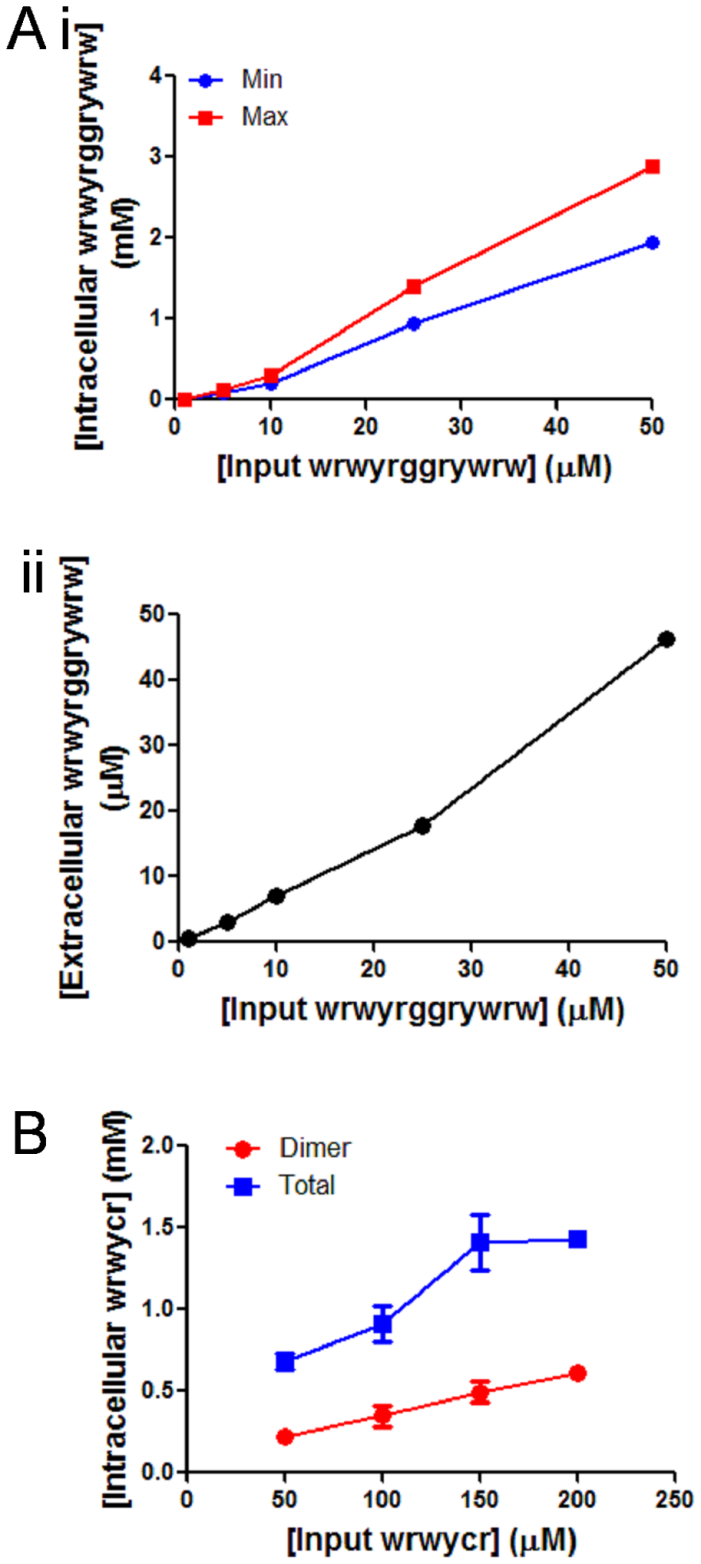
Determination of the intracellular concentration of wrwycr. **A:** PC3 cells were independently treated with 50, 100, 150, 200 µM of wrwycr for 48 h. (i): Intracellular concentration was calculated as described in Materials and Methods. Final intracellular peptide concentrations (in mM) were calculated taking into account two reported volumes for the cells: the minimum concentration (blue line) denotes wrwyrggrywrw concentration based on a cell volume of 2.4 pl and the maximum concentration (red line) denotes the concentration based on a cell volume of 1.6 pl. (ii): Extracellular concentration was calculated from the peptide left in the media and graph against input concentration of wrwyrggrywrw in X-axis. **B**: HeLa cells were independently treated with 50, 100, 150, 200 µM of wrwycr for 24 h, cell lysates were fractionated using HPLC, and the area under the curve of the peptide-dependent peaks was calculated as described in Materials and Methods.

To investigate whether peptide wrwycr or wrwyrggrywrw accumulated within cells, the concentration of the unlabeled and unmodified peptides was directly determined using an HPLC-based method. PC3 or HeLa cells were treated with different concentrations of the wrwycr or wrwyrggrywrw for 48 h, and the cells were separated by centrifugation from the media, which was examined separately for the presence of the peptide using a similar HPLC method [30]. The cells were washed several times to remove peptide in the media or peptide loosely associated with cell surfaces. Cells were then lysed with a detergent-containing buffer and the soluble fraction was obtained by centrifugation and analyzed using HPLC. The peptide concentrations in the cell lysates were determined using a standard curve as described in Materials and Methods. Cell membranes are not included in the soluble fraction analyzed, and hence it is likely that most of the peptide observed by HPLC is intracellular. The concentration of both wrwycr and wrwyrggrywrw increased as cells were incubated with increasing concentration of the peptide (Figure 2). The concentration of both peptides in the cell lysates increased with increased input concentration of the peptide (Figure 2Ai and 2B). In parallel, we calculated the concentration of peptide in the media, and found that, at several input concentrations, the media had roughly 5–10% less peptide (Figure 2Aii). This could be due to some peptide binding to serum proteins present in the media and thus becomes unavailable to the cells. On the other hand, both wrwycr and wrwyrggrywrw become more concentrated inside the cells, a phenomenon also observed in U2OS cells by Sukanya Patra [unpublished data] and in J774A.1 cells [30]. For example, at 25 µM input wrwyrggrywrw dose, the intracellular concentration reached nearly 1 mM (Figure 2Ai). At high peptide concentration, where cells are more likely to die than replicate, actual intracellular concentrations may have been underestimated since the intracellular peptide concentrations with treatment were calculated based on the number of cells at the beginning of the experiment. Thus cells appear to concentrate the peptide, perhaps because it binds to cellular constituents and is trapped inside. Previously, when fluorescently labeled peptide was used to track the localization of the peptide, rhodamine-labeled peptide but not rhodamine alone was observed inside cells (supplemental data in reference [30]). One caveat of our quantification is that, although the membranes are separated from the soluble fraction during our preparation of cell lysates, some of the membrane-associated peptide may have been solubilized by the detergent and may contribute to the apparent intracellular concentration of peptide, leading to an overestimate of the intracellular peptide. The rhodamine-labeled peptide had no obvious preference for membranes, however [30]. The mechanism by which the peptide is internalized within the cells is not clear. Our previous studies have indicated that peptide uptake is an active process, based on the observations that lowering the temperature from 37°C to 4°C as well as the use (at 37°C) of the PI3K inhibitor wortmannin both reduced the uptake of the peptide, suggesting that endocytosis plays a role in the uptake of the peptide [30].

### Peptide-induced cell death is independent of apoptosis

**Figure 3:**
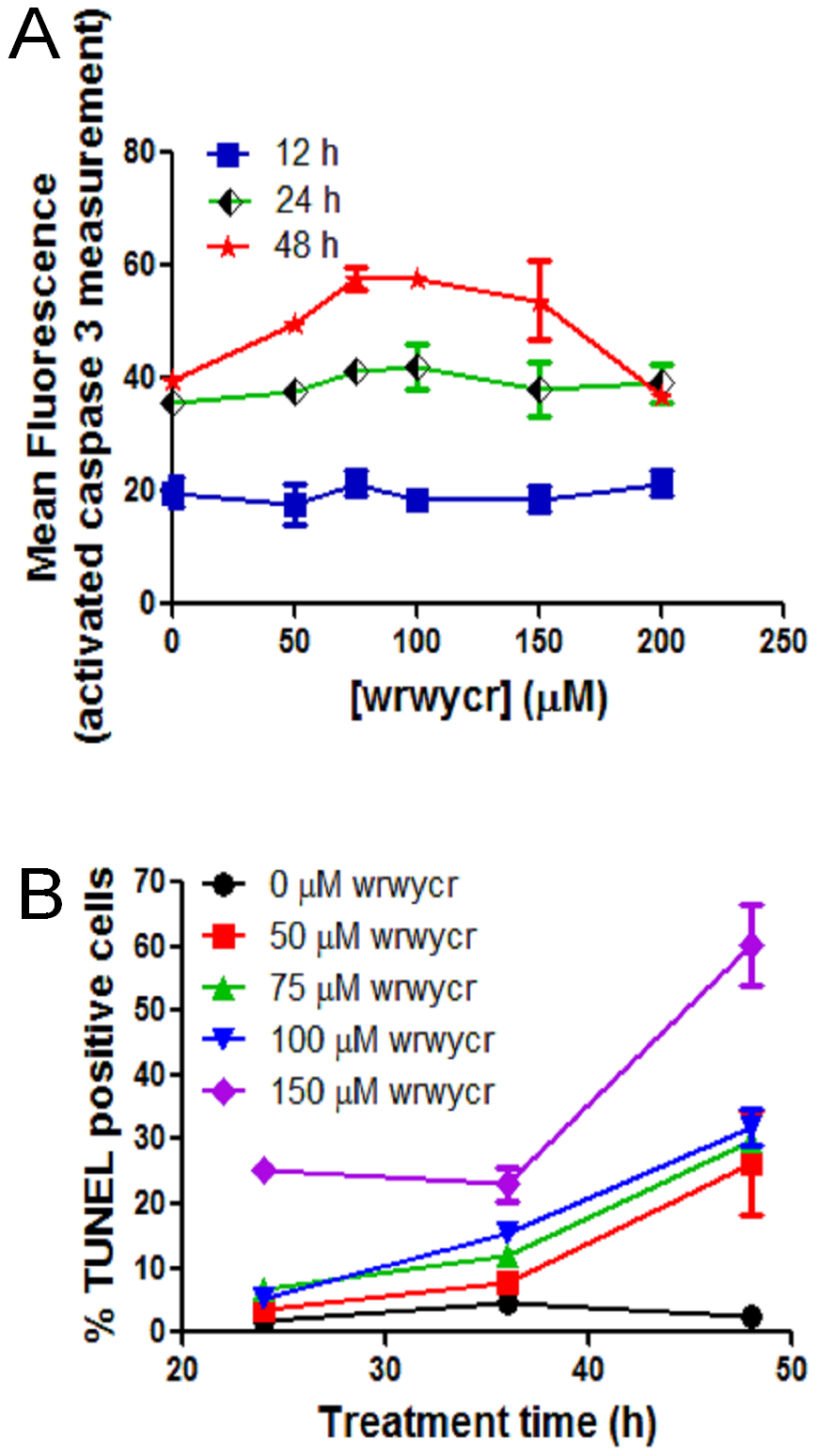
Investigating markers of apoptosis. **A:** PC3 cells were treated with 50, 75, 100, 150, 200 µM of wrwycr for 12, 24 or 48 h and examined for caspase-3 markers. The reading from spectrophotometer was normalized to the protein concentration of each sample and mean fluorescence in Y-axis was plotted after dividing the fluorescence from each sample by the corresponding protein concentration at OD_595_. **B**: PC3 cells were treated as specified with wrwycr for 12, 24 and 48 h, and examined for DNA breaks using the TUNEL assay. A significant increase in TUNEL positive cells was observed with all the different doses of wrwycr treatment after 48 h, as determined by a two-way ANOVA using Bonferroni post-test analysis.

The decrease in cell survival with peptide treatment led us to investigate the mode of cell death, specifically apoptosis. Caspase-3 is an effector caspase that is activated as a result of proteolytic processing by initiator caspases. Active caspase-3 is a well-known executioner of apoptosis. To investigate if peptide-induced cell death occurs via apoptosis, we examined caspase-3 activation as a late marker of apoptosis in PC3 cells treated with wrwycr over a time course of 12–48 h (Figure 3A). Although Figure 1 showed that cell viability decreases with increasing peptide treatment, there was no significant activation of caspase-3 with increasing doses of wrwycr (50–200 µM) compared to the DMSO control (Figure 3A). However, caspase-3 activity increased significantly with time, even in DMSO-treated cells.

Apoptosis is associated with genome fragmentation, and thus we tested if DNA breakage is associated with wrwycr-induced cell death. We performed a terminal deoxynucleotidyl transferase (TUNEL) assay and found that as high as 65% of the PC3 cell population is TUNEL positive after 48 h treatment with 150 µM wrwycr treatment (Figure 3B).

**Figure 4:**
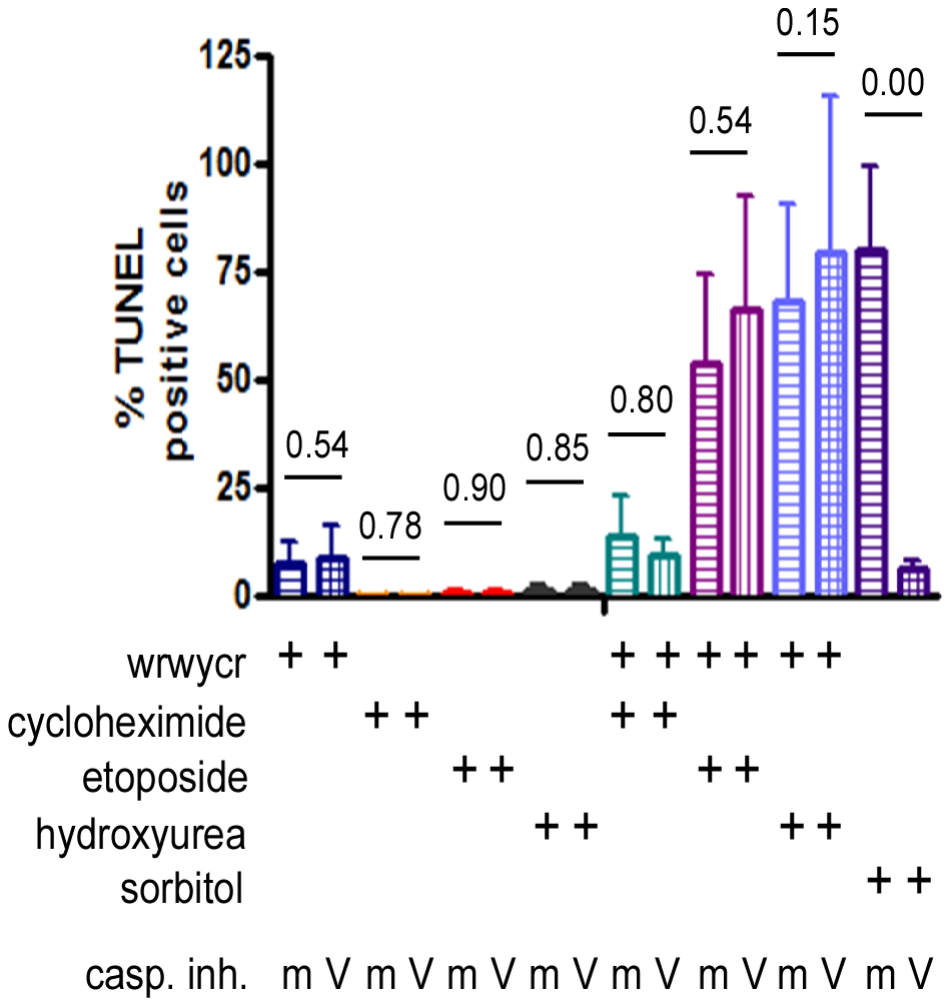
Effects of wrwycr on DNA breaks in HeLa cells. HeLa cells were incubated with the indicated treatments for 24-VAD-fmk (V) and a mock inhibitor z-FA-fmk (m) were used in conjunction with various treatments to determine if apoptosis contributes significantly to the accumulation of DNA breaks. The p-values reported are the results of Wilcoxon exact two-tailed test.

To test whether the TUNEL signal was dependent on activation of apoptosis, we did the assay in HeLa cells in the presence of the caspase inhibitor z-VAD-fmk, the broad spectrum caspase inhibitor, or the mock inhibitor z-FA-fmk [39], to assess the contribution of apoptotic events to the TUNEL signals detected after peptide treatment. In comparison to PC3 cells, only 13% of HeLa cells treated with 100 µM wrwycr were TUNEL-positive (Figure 4). No significant difference in TUNEL positive wrwycr-treated cells was seen in the presence of the caspase inhibitor versus the mock inhibitor. As a control, we used 400 mM sorbitol treatment, which is known to induce apoptosis by osmotic shock; in this case, nearly 80% of cells incubated with the mock inhibitor were TUNEL-positive, while fewer than 10% of the z-VAD-fmk inhibitor-treated cells were TUNEL-positive (Figure 4). This strongly suggests that the TUNEL signals detected in the wrwycr-treated cells were due to an activity of the peptide rather than to the induction of apoptosis-dependent cleavage of DNA.

**Figure 5:**
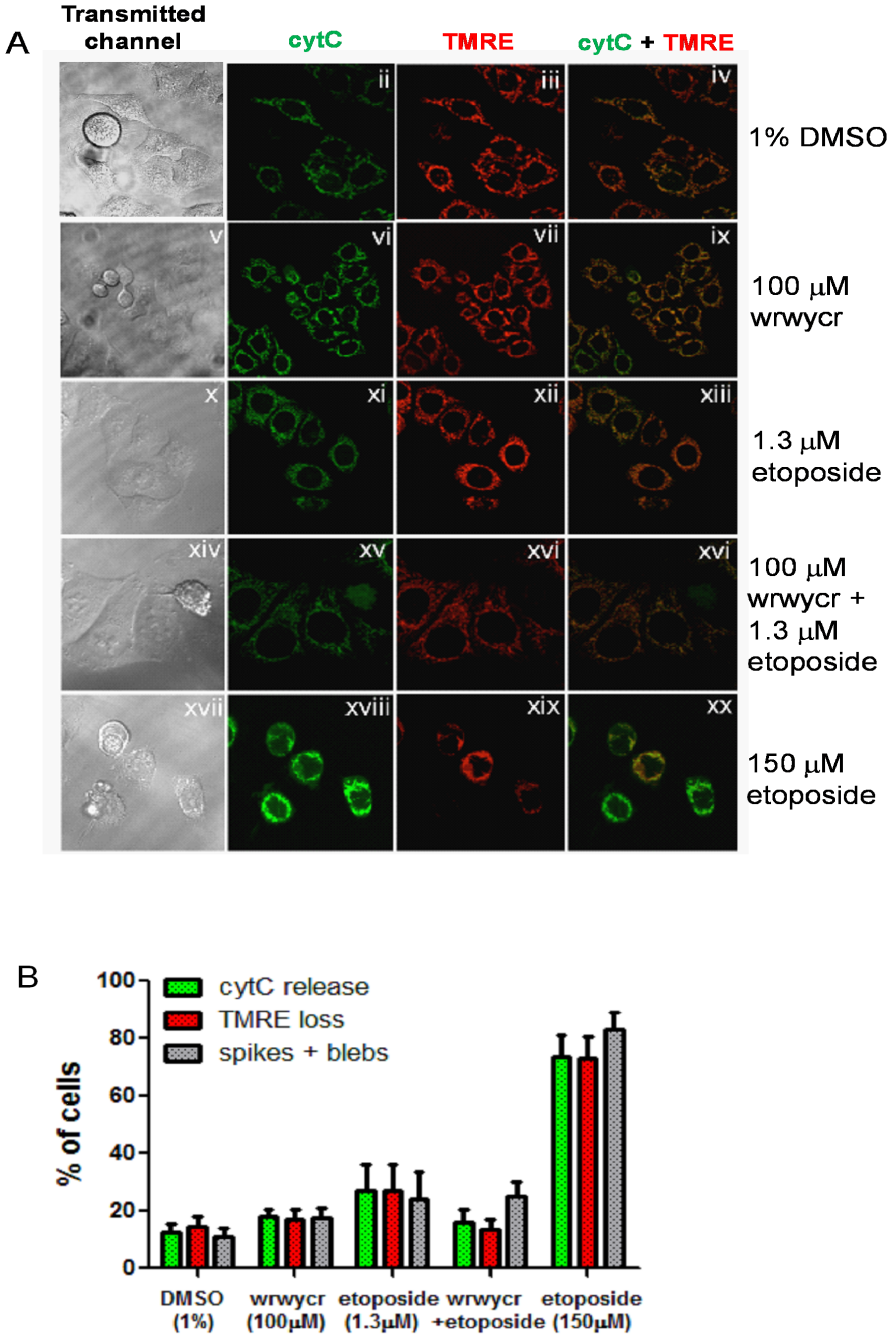
wrwycr treatment with or without etoposide does not induce significant apoptosis. **A:** Confocal microscopy images showing the transmitted channel (1^st^ column), cytochrome C-GFP (2^nd^ column), TMRE (3^rd^ column) and the overlay of GFP and TMRE channel (4^th^ column) followed by 24 h treatment. **B**: % Apoptotic cells scored by the release of cytochrome C (green bar), loss of TMRE (red bar), and typical morphological features of apoptosis such as spikes or blebs (grey bar).

Both prior approaches to detect apoptosis were based on the activities of caspases, which carry out the late stage events of apoptosis. In order to test earlier hallmarks of apoptosis, we used HeLa cells stably transfected cell derivatives with a cytC-GFP fusion protein (hTog1) [40]–[42]. The intracellular distribution of the cytochrome C (cytC) protein during various stages of apoptosis [40]–[42] was examined by microscopy. Co-staining the hTog1 cells with a fluorescent probe, TMRE dye, that partitions into the mitochondrial matrix in response to the creation of Δψm, reported the status of the mitochondrial membrane potential [43]. We observed little or no cytochrome C release or loss of TMRE occurring in peptide wrwycr-treated cells, indicating that they have not initiated apoptosis (Figure 5). This is even more evident by a tight overlay of the two channels (Figure 5 iv,ix,xiii,xvi). In contrast, in cells treated with 150 µM etoposide, a concentration reported to cause apoptosis via DNA damage in HeLa cells [44], cytC and TMRE staining do not coincide, indicating loss of mitochondrial membrane potential and release of cytC by the positive control treatment (Figure 5A panel xx).

### Peptide wrwycr induces DNA-damage sensors

**Figure 6:**
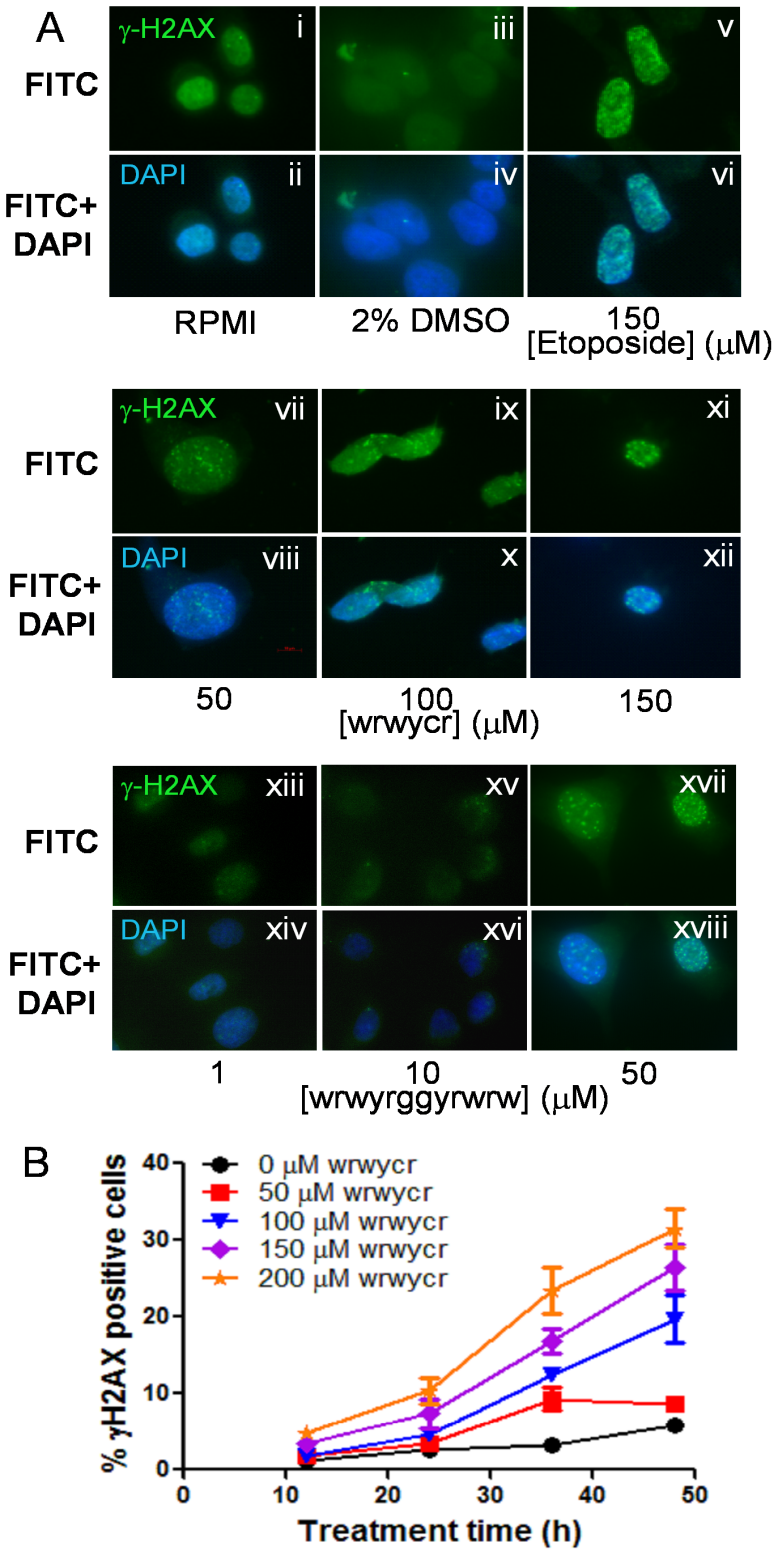
wrwycr-induced γH2AX foci. **A:** PC3 cells were treated with RPMI (**i–ii**), DMSO (**iii–iv**), 150 µM etoposide (**v–vi**), or wrwycr at 50 (**vii–viii**), 100 (**ix–x**), 150 (**xi–xii**) µM or wrwyrggrywrw at 1 (**xiii–xiv**), 10 (**xv–xvi**) or 50 (**xvii–xviii**) µM for 48 h. Etoposide was used as the positive control. The green γH2AX foci are visible within the nucleus. **B**: γH2AX foci in PC3 cells were determined over time (12, 24, 36, 48 h) and quantified using flow cytometry. The γH2AX positive population was gated with respect to FITC-conjugated isotype control antibody. The differences in % γH2AX positive cells are statistically significant for all wrwycr doses between 100–200 µM compared to DMSO after 36 and 48 h of treatment as determined by two-way ANOVA using Bonferroni post test analysis.

To further characterize peptide wrwycr- or wrwyrggrywrw-induced DNA damage in cells, we examined the activation of H2AX, a variant of H2A histone protein activated by phosphorylation at Ser139 following ds DNA damage. H2AX is considered to be the first sensor molecule that is phosphorylated at Ser 139 residue, forming γH2AX, in response to DNA damage, most likely DSBs [45], [46]. Formation of γH2AX foci at the sites of DNA damage was monitored by using a fluorescent antibody against the phosphorylated Ser139 of H2AX. Cells were treated with 50–200 µM of wrwycr or 1–50 µM of wrwyrggrywrw for 48 h and were evaluated for the accumulation of γH2AX foci in peptide-treated and untreated cells as well as in etoposide-treated cells, as a positive control (Figure 6A). Compared to untreated and DMSO-treated cells, treatment with etoposide (150 µM) caused marked accumulation of γH2AX-positive cells (Figure 6A v–vi). Increasing wrwycr doses (50–150 µM) or wrwyrggrywrw (1–50 µM) have a similar effect (Figure 6A vii–xviii). To further characterize and better quantify the appearance of γH2AX positive cells over time, we treated PC3 cells with wrwycr for 12, 24, 36 and 48 h. The fraction of γH2AX positive cells increased over time with increasing dose of peptide wrwycr (Figure 6B). Treatment of cells with 200 µM of peptide resulted in 2–5% of γH2AX positive cells between 12–24 h, which increased to 20–35% after 36–48 h of treatment a peptide-dependent, dose-dependent and time-dependent increase in DSBs.

**Figure 7:**
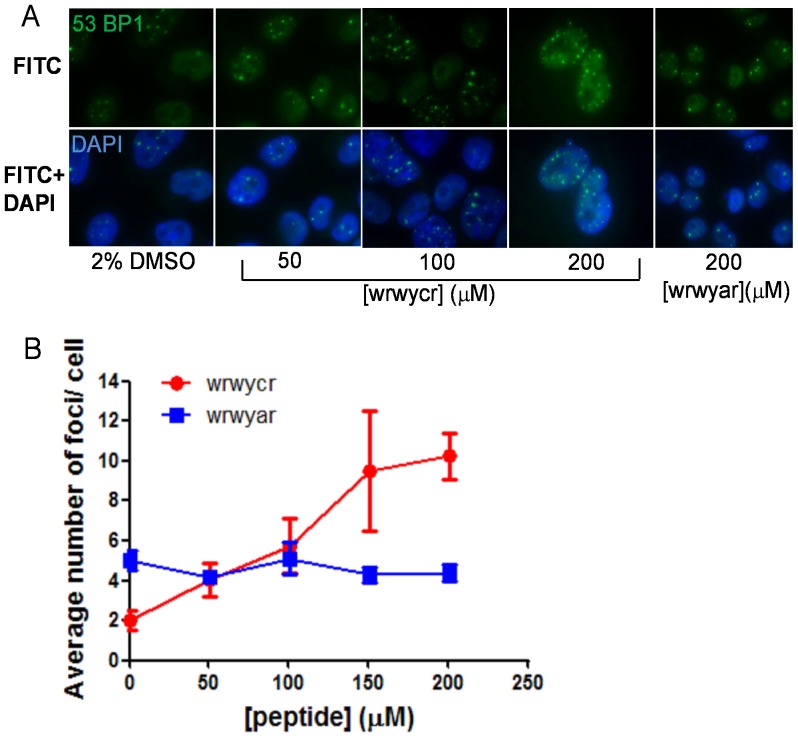
Treatment with wrwycr elicits 53BP1 focus formation in the PC3 nucleus. PC3 cells were treated as specified for 48**A**: Fluorescence microscopy images of 53BP1 foci and merged images with the nuclear stain DAPI and 53BP1 foci in cells treated with 50–200 µM wrwycr. **B**: Foci formed in cells treated as specified were counted and the average number of foci per cell was calculated for 35–79 cells from 2 independent experiments. Significant accumulation of 53BP1 was observed with 200 µM wrwycr vs. DMSO, as determined by one-way ANOVA using Bonferroni post test analysis.

We confirmed that peptide wrwycr induced DNA damage by examining the accumulation of 53BP1 protein foci, which depends on the phosphorylation of γH2AX by ATM [47] and is associated with the histone H4 protein at the site of DNA damage [48]. We treated PC3 cells with several doses of wrwycr for 48 h. Our data showed that wrwycr treatment leads to the accumulation of 53BP1 protein in a dose-dependent manner after 48 h, evident from the average number of 53BP1 foci formed in each cell (Figure 7A). Treatment of PC3 cells with the control peptide wrwyar did not lead to accumulation of 53BP1 foci after 48 h, confirming that induction of DNA damage, most likely breaks, within PC3 cells depends on treatment with the dimeric wrwycr (Figure 7B).

### Peptide wrwycr arrests cellular progression through the cell cycle

**Figure 8:**
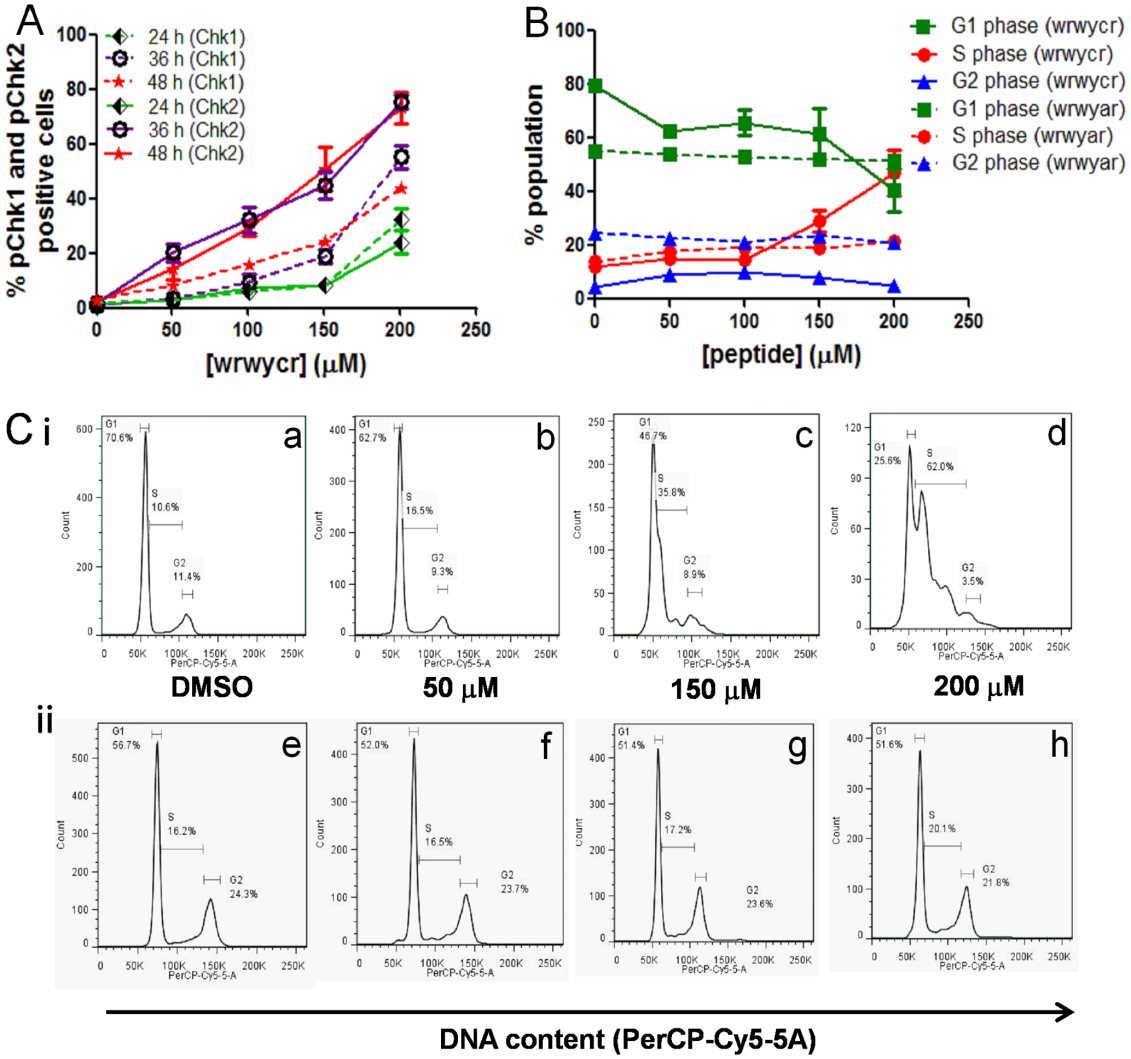
Treatment with wrwycr interferes with the PC3 cell cycle over time. PC3 cells treated with 50, 100, 150, 200 µM wrwycr or 200 µM wrwyar for 24, 36 or 48 h. **A**: Phosphorylation of checkpoint proteins was analyzed using BDFacs Diva software. Significant increases in the fraction of pChk1^+^ cells were observed with 150 and 200 µM wrwycr, and in the fraction of pChk2^+^ cells with 100,150 and 200 µM wrwycr, after 36 h of treatment, as shown by two-way ANOVA using Bonferroni post test analysis. **B**: Cell cycle analysis of wrwycr or wrwyar- treated PC3 cells after 48 h. Graphical representation of the percentage of population in each of the phases of cell cycle done at 48 h are from 2 independent experiments, each with duplicate or triplicate independent samples. A significant increase in the fraction of S-phase cells was observed after treatment with 200 µM wrwycr compared to treatment with DMSO, as determined by one-way Anova using Bonferroni post-test analysis. **C**: Histograms of the DNA content in (**i**) wrwycr (**a–d**) or (**ii**) wrwyar- treated (**e–h**) cells at different peptide concentrations (0–200 µM). Cells in the G2 peak have twice the DNA content of G1. Cells in transition to G2 from G1 are considered to be in S-phase. **C**: Graphical representation of the percentage of population in each phase at 48 h from 2 independent experiments, each with duplicate or triplicate independent samples. A significant increase in the fraction of S-phase cells was observed after treatment with 200 µM wrwycr compared to treatment with DMSO, as determined by one-way ANOVA using Bonferroni post-test analysis.

Given that cells accumulate wrwycr-dependent DNA damage, we investigated the activation of downstream DNA repair signaling pathways. We first examined the phosphorylation of the cell cycle checkpoint proteins Chk1 and Chk2, known components of the DNA damage checkpoint control pathway [49]. The Chk1 serine kinase is phosphorylated at Ser317 and Ser345 by the ATR protein (ATM and Rad3-related), while the Chk2 serine threonine kinase is phosphorylated at Thr68 by ATM upon DSB accumulation [50]. Phosphorylated Chk1 activates the G2/M checkpoint to prevent cells from entering mitosis, while activated Chk2 prevents the passage of damaged cells from G1 to S phase of the cell cycle. Chk1 and Chk2 phosphorylation at Ser345 and Thr68 respectively were investigated following treatment with different concentrations (50–200 µM) of wrwycr after 24, 36 or 48 h using flow cytometry. Relatively low levels of activated Chk1 and Chk2 molecules were found after 24 h except for the highest peptide dose of 200 µM (Figure 8A). After 36 or 48 h, the fraction of cells with activated Chk1 and Chk2 increased both with time and with dose of peptide. Chk2 was activated earlier and to a greater extent than Chk1 (Figure 8A).

Our analyses established that wrwycr treatment led to accumulation of DNA damage, including DNA breaks, and to the activation of the checkpoint control kinases, Chk1 and Chk2. Based on these observations, we predicted that the peptide would induce cell cycle arrest. To test this prediction, we examined the cell cycle distribution of PC3 cells after 48 h (Figure 8B) or 72 h (Figure S1) of peptide treatment. We measured the DNA content in the cellular population after serum starving PC3 cells for 48 h to synchronize the entire population in G_0_ phase. Cells were then cultured in growth media supplemented with serum and with 100–200 µM of wrwycr, wrwyar, or DMSO for 48 h or 72 h. For DMSO or wrwyar treated samples, we found two peaks corresponding to cells in G2 having twice the DNA content as the cells in G1 (Figure 8B ii). With increasing doses of wrwycr (150–200 µM), a new peak emerged, representing cells accumulating in S-phase (Figure 8B i). The percentage of the S phase cellular population significantly increased from 14% with vehicle control (DMSO) treatment to 50% with 200 µM wrwycr treatment at 48 h or 72 h (Figure 8C and Figure S1), suggesting that wrwycr is arresting cells in S-phase. The control peptide wrwyar did not exert this effect.

### Peptide shows additive or synergistic effects with chemotherapeutic drugs that cause DNA damage

The experiments above showed that peptide wrwycr accumulates DSBs in an apoptosis-independent manner and arrests the cells' passage beyond S-phase. Therefore, we examined whether the effects of peptide wrwycr are potentiated by other chemotherapeutic agents. To this end, we combined peptide wrwycr with chemotherapeutic agents that lead to DSBs (doxorubicin or etoposide) or to replication fork collapse (hydroxyurea (HU)), or with the mitotic spindle inhibitor docetaxel or the protein synthesis inhibitor cycloheximide, and tested the effects of the combination treatment on PC3 and HeLa cells. In each case, the effects of each agent were first examined individually to identify sub-lethal doses of these agents. Subsequently the effects of combination treatments were determined.

**Figure 9:**
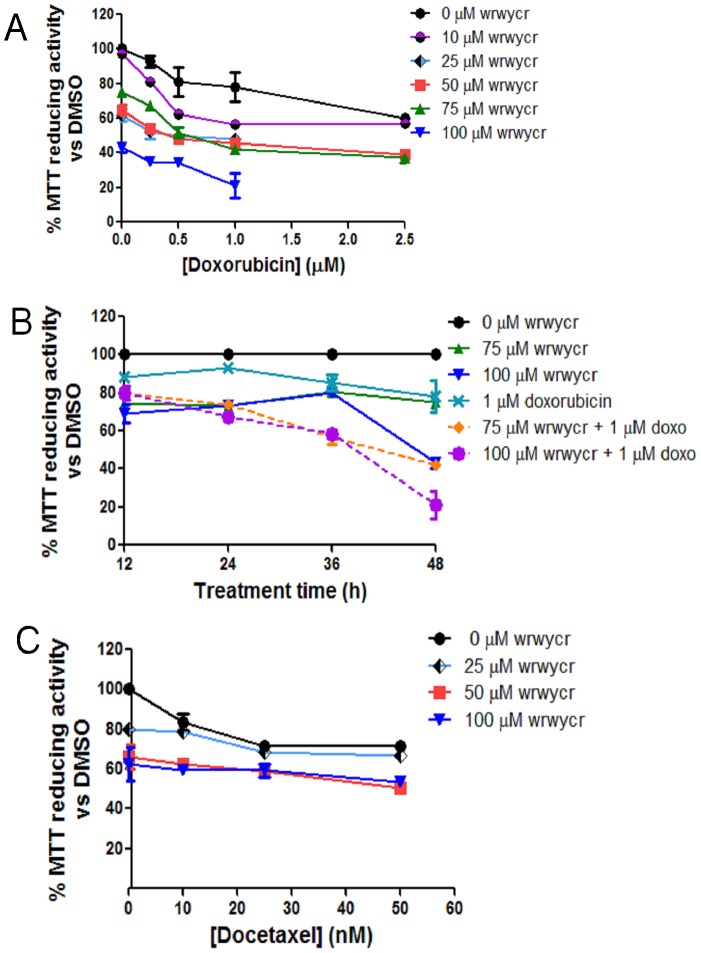
MTT assays of PC3 cells treated with a combination of wrwycr and doxorubicin or docetaxel. **A:** PC3 cells were treated with 10, 25, 50, 75 and 100 µM wrwycr and/or with 0.25, 0.5, 1 or 2.5 µM doxorubicin for 48 h. **B**: Time course of treatment with 75 or 100 µM of wrwycr and 1 µM doxorubicin either alone or in combination for 12–48 h. **C**: PC3 cells treated with 0, 25, 50 and 100 µM wrwycr and/or with 10, 25, or 50 nM of docetaxel. MTT activity was converted to % survival after normalizing to the activity of DMSO-treated cells.

PC3 cells were treated with sublethal doses of the peptide (25–100 µM) either alone or in combination with doxorubicin (0.25–25 µM) for 48 h. Combination treatments revealed that MTT reducing activity decreased additively or more than additively when peptide and doxorubicin were combined, compared to that with single treatments (Figure 9A). Treatment of PC3 cells with 2.5 µM of doxorubicin in combination with 75 µM of wrwycr for 48 h had an additive effect on cell survival, and combining 2.5 µM of doxorubicin with 100 µM of wrwycr showed a greater than additive effect. For example, 2.5 µM of doxorubicin treatment alone decreased cell survival to 73% and 100 µM of peptide alone decreased it to 75% (Figure 9A). If the combination treatment were additive, cell survival would have been ∼54% viability (0.73×0.75 = 0.5475); the combination treatment decreased cell survival to 42% (Figure 9A). The effect was time dependent: it was not evident at 12 or 24 h but became evident at 36 h, Whereas treating cells either with 1 µM of doxorubicin or with 75 or 100 µM of wrwycr reduced cell survival to 80%, the combined treatments reduced cell survival additively, to 60% (Figure 9B). This suggested that wrwycr-induced cell death is potentiated by DNA damaging agents, similar to our observations in bacteria [25].

On the other hand, co-treatment with docetaxel elicited a completely different outcome. When PC3 cells were treated for 48 h with 10–50 nM of docetaxel, cell survival decreased from 90% to 80%, while 25–100 µM of wrwycr reduced cell survival from 80% to 60% (Figure 9C). The combination treatment could not decrease the cell survival any further than 58% with the combination treatment (Figure 9C) suggesting that the effects of wrwycr were not either additive or synergistic with those of docetaxel. Thus, while doxorubicin potentiated the effect of the peptide (and vice versa), docetaxel did not.

To further test the effects of the peptide in conjunction with other chemotherapeutic drugs, we treated HeLa cells with a combination of peptide and etoposide or HU; the latter inhibits production of deoxyribonucleotides and leads to collapse of replication forks. Independent treatment with 1.3 µM etoposide or 10 mM hydroxyurea resulted in fewer than 2% TUNEL-positive cells. When co-treated with the peptide and cycloheximide, an inhibitor for protein synthesis, nearly 16% of the cells became TUNEL-positive, showing the additive effect of the two treatments. However, when HeLa cells were co-treated with peptide wrwycr and either etoposide or HU, roughly 60% and 80% of the cells, respectively, were TUNEL-positive (Figure 4A), much higher than would be expected if the effects were simply additive (∼15%). These data further supported our hypothesis that peptide wrwycr interacts with DNA repair intermediates and causes the accumulation of DNA breaks in mammalian cells. And, like the effects of the peptide on its own, the damage caused by the combination of peptide and etoposide or HU was independent of caspase-3 activation (Figure 4B).

**Figure 10:**
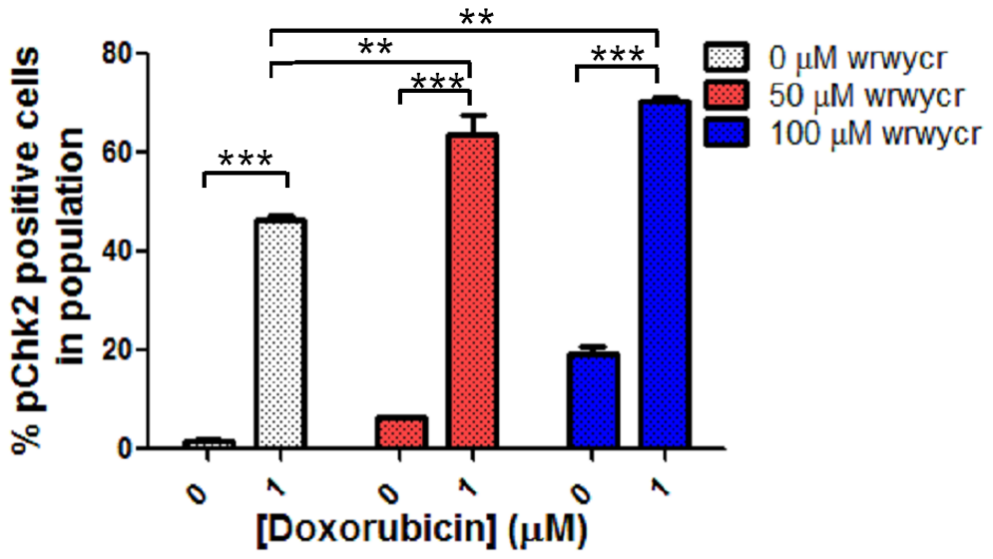
Co-treatment of PC3 cells with doxorubicin and wrwycr significantly increases the population of pChk2^+^ cells compared to corresponding individual treatments. PC3 cells were treated with 50, 100 µM wrwycr and 1 µM doxorubicin for 48 h either alone or in combination. Significance was determined with one-way ANOVA using Bonferroni post-test analysis. *** indicates p<0.001, ** indicates p<0.01.

We also tested the activation of Chk2 proteins in PC3 cells co-treated with wrwycr and doxorubicin to examine if these compounds potentiate each other's activities. About 5% cells had activated Chk2 when treated with peptide wrwycr at 50 µM concentration on its own, whereas 50% of the cellular population had activated Chk2 when treated with 1 µM doxorubicin on its own. When co-treated with wrwycr and doxorubicin, over 60% of the cells were pChk2-positive (Figure 10), significantly higher than with either of the single treatments. Increasing the concentrations of both wrwycr and peptide enhanced the effect on Chk2 activation. The intensity of the mean fluorescence of the Chk2 population upon combination treatment was also found to be significantly high than the doxorubicin or wrwycr treatments alone (Figure S2).

### Peptide wrwycr does not induce ER stress

In bacteria, peptide wrwycr induced envelope stress in bacteria. The envelope stress response is activated by ethanol and heat treatment as well as treatment with some antibiotics, and induces the expression of chaperone proteins and proteases, among others, that clear misfolded proteins from the periplasm [51]–[53]. Strains with mutations in the chaperone proteins were hypersensitive to peptide wrwycr [54]. Because bacterial envelope stress shares some similarities with the ER stress response in eukaryotes, we examined whether the ER stress response was activated in HeLa cells [55]. We studied the induction of the GRP78 promoter, a reporter for the ER stress response, upon peptide treatment and used the luciferase gene expression from the GRP78 promoter as an indicator of ER stress. Peptide wrwycr did not induce expression of the GRP78 promoter, whereas tunicamycin, a known inducer of ER stress, elicited its expected response (Table 2).

**Table 2:** wrwycr does not induce the ER stress response in HeLa cells.

Treatments	pGRP78^a^		pSV40^b^		pGRP78/pSV40^d^
	Units	Fold change	Units	Fold change	
DMSO	118163±1459		60627±3016		
100 µM wrwycr	184755±11572	1.53±0.09	112162±4444	1.9±0.17	0.84±0.1
150 µM wrwycr	214642±18292	1.8±0.1	135441±32099	2.9±0.05	1.02±0.44
200 µM wrwycr	282941±27749	2.4±0.2	165928±1165	2.7±0.09	0.87±0.06
wkhyny	62097±1715	0.50±0.02	56685±1657	0.9±0.02	0.55±0.01
Tunicamycin^c^	425255±16205	7.9±0.09	4606±362.1	0.4±0.02	20.0±1.36

a plasmid DJT208 carries the GRP78 promoter (pGRP78) which drives the ER-stress response elements ERSE 1, ERSE 2 and ERSE 3 upstream of the luciferase gene.

b plasmid pGL2P carries the luciferase gene driven by the SV40 promoter (pSV40).

c Tunicamycin, a known inducer of ER stress, was used as a positive control.

d ER stress was calculated as the fold increase of luciferase gene expression from the GRP78 promoter over that from the SV40 promoter of pGL2P for the different concentrations of the peptide over DMSO or over the media in the case of TM.

## Discussion

Peptides wrwycr and and its single chain analog wrwyrggrywrw prevent HJ resolution by several non-homologous and structurally unrelated HJ-resolving proteins, and bind HJs and replication forks [19], [20], [22], [37]. In this study, we demonstrated that peptide wrwycr and wrwyrggrywrw induce tumor cell death, using MTT, Live/Dead and colony-forming assays, and accumulation of DSBs. We further showed that induction of cell-death is coincidental with the induction of the DNA damage response and arrest of the cell cycle in S phase. Unusually, peptide-induced cell death is not dependent on caspase 3-regulated apoptotic pathways. Because we observed similar effects with wrwycr and wrwyrggrywrw, we continued working predominantly with wrwycr for the rest of the study.

Cancer cells incur higher levels of single and double stranded (ds) DNA breaks due to the higher metabolism and uncontrolled replication [56]. Previous studies have shown that the expression of genes related to HRR is elevated 2–5 fold in prostate cancer cells over the level in normal prostate epithelial cells [57]. DNA repair pathways are increasingly being explored as targets for chemotherapeutic drugs [58]. HJs and other branched DNA intermediates such as D-loops formed as intermediates during homologous recombination are predicted to be the targets of our peptide. Peptide treatment of *E. coli* and *Salmonella enterica* cells led to the accumulation of single and double stranded DNA breaks, accumulated HJs, and interfered with chromosome segregation. These observations raised the intriguing possibility that the peptide may be cytotoxic in cells with higher levels of DNA damage and with greater dependence on DNA repair.

Indeed, both peptide wrwycr and the single peptide chain mimic of the wrwycr dimer, wrwyrggrywrw, were cytotoxic to several tumor cell lines, some of which were more sensitive to the peptides than others. We do not know the basis of this difference, although it does not correlate with the p53 status of the cell lines. Peptide wrwycr treatment caused the accumulation of DNA breaks in a dose and time dependent manner, as evident from TUNEL assays, as well as increased formation of γH2AX foci (also shown for wrwyrggrywrw) and 53BP1 foci. Formation of γH2AX foci is usually transient, and foci dissipate upon dephosphorylation by phosphatases or by replacement of γH2AX by unmodified H2A in the presence of an efficient repair system [59]. Persistent γH2AX foci either represent irreparable DSBs or rejoined ds breaks without restoration of chromatin structure [59]. γH2AX accumulation leads to activation of downstream kinases, ATM and ATR, which in turn activates the checkpoint proteins, Chk1 and Chk2. Indeed we observed the activation of Chk1 and Chk2. In consequence, peptide wrwycr treatment arrested 50% of the PC3 population in S-phase even after 72 h.

Peptide wrwycr-induced S phase arrest in PC3 cells was also evident after co-treatment with the peptide and other chemotherapeutics. Peptide wrwycr potentiated the effect of etoposide, doxorubicin, and HU, all of which act during S phase. In contrast, the mitotic inhibitor docetaxel, which acts in M-phase, did not elicit additive effects with peptide wrwycr – presumably any cell not stalled in S phase by peptide wrwycr would be blocked in M phase by docetaxel.

A major challenge of cancer treatment is drug delivery. The intact cell membrane protects the cellular components from its surroundings, restricting hydrophilic molecules from entering the cell and allowing only small molecules to cross the membrane. The presence of hydrophobic and basic amino acid residues in peptide wrwycr probably helps it cross the cancer cell membrane more efficiently than normal cells, similar to the cell penetrating peptides (CPPs) [54]. The intracellular concentration of both wrwycr and wrwyrggrywrw in HeLa and PC3 cells, respectively increased in a dose-dependent manner (Figure 2). The uptake of peptide wrwycr in U2OS cell lines is 3× greater than in non-tumor derived IMR 90 cells [Sukanya Patra Ph.D dissertation]. Exactly how the peptide crosses the membrane is not yet clear. A class of CPP, known as the non-amphipathic CPPs, is rich in cationic amino acids and interacts with anionic amino acids present in the phospholipid membrane proteins [60]–[62]. Cancer cells are documented to have higher membrane potential and higher concentration of anionic phospholipids on their outer membrane leaflets [63] and thus can take up CPPs more efficiently than normal cells. The combination of aromatic/hydrophobic amino acids present in peptide wrwycr is similar to the structure of non-amphipathic CPPs. This similarity may confer the apparent selective advantage to peptide wrwycr with respect to uptake by cancer cells compared to normal cells [30].

Further studies are necessary to define the exact mechanism(s) of peptide wrwycr-dependent cytotoxicity. Prolonged cell cycle blockage did not activate apoptosis in either PC3 or HeLa cells, both of which are p53-deficient. Caspase-independent DNA fragmentation has been shown previously, where mitochondrial endonuclease G translocates to the nucleus upon apoptotic signaling and causes DNA fragmentation in a caspase-independent manner [64]. However, no change in mitochondrial membrane potential was found in HeLa cells, indicating that wrwycr-induced DNA DSBs are independent of the activity of mitochondrial endonucleases. Other mechanisms of cell death may occur, for example necrosis, where depletion of intracellular ATP results in swelling and blebbing of the plasma membrane, ultimately leading to cell death [65], but this is not entirely consistent with the observed morphological changes in either PC3 or HeLa cells. Other possibilities we are considering, based on the *in vitro* properties of the peptide, are DSB accumulation due to the binding of wrwycr to DNA repair intermediates such as HJ and collapsed replication forks. Re-replication followed by the collision of forks with the sites of damage would result in amplification of the DSB signal. Studies testing this hypothesis are in progress.

In conclusion, our study demonstrated the potential utility of peptide wrwycr, and compounds with similar activities, as an anti-tumor therapeutic agent. For many cancers, treatment options are very limited. For example, in the case of prostate cancer, first line treatment chemotherapeutics used alone or in combination with drugs such as prednisone against metastatic cancer resulted in only a modest improvement in patient survival [66]. Development of new treatments, especially those that synergize with the physiology of specific tumors, and treatments that enhance the efficacy of currently used chemotherapeutics should be very desirable.

## Supporting Information

Figure S1 **Interference with the PC3 cell cycle by wrwycr treatment for 72 h.** Graphical representation of PC3 cells treated with wrwycr for 72 h from cell cycle analysis performed as the analysis shown in Figure 8B. A significant increase in the fraction of S-phase cells was observed after treatment with 200 µM wrwycr compared to treatment with DMSO, as found by one-way Anova using Bonferroni post-test analysis.
(TIF)

Figure S2 **Mean Fluorescence intensity of pChk2 population increases with combination treatment of doxorubicin and wrwycr.** PC3 cells were treated with 50, 100 µM wrwycr and/or 1 µM doxorubicin for 48 h and analyzed for the activation of Chk2, as described in Materials and Methods. Significance was determined with a one-way Anova using Bonferroni post-test analysis. *** indicates p<0.001, ** indicates p<0.01 and * indicates p<0.05.
(TIF)
