# Supplementary figures and images for: Tumor Cell Death Mediated by Peptides That Recognize Branched Intermediates of DNA Replication and Repair
Source: PLoS One. 2013 Nov 14;8(11):e78751. doi: 10.1371/journal.pone.0078751 (PMC3828334; doi:10.1371/journal.pone.0078751)

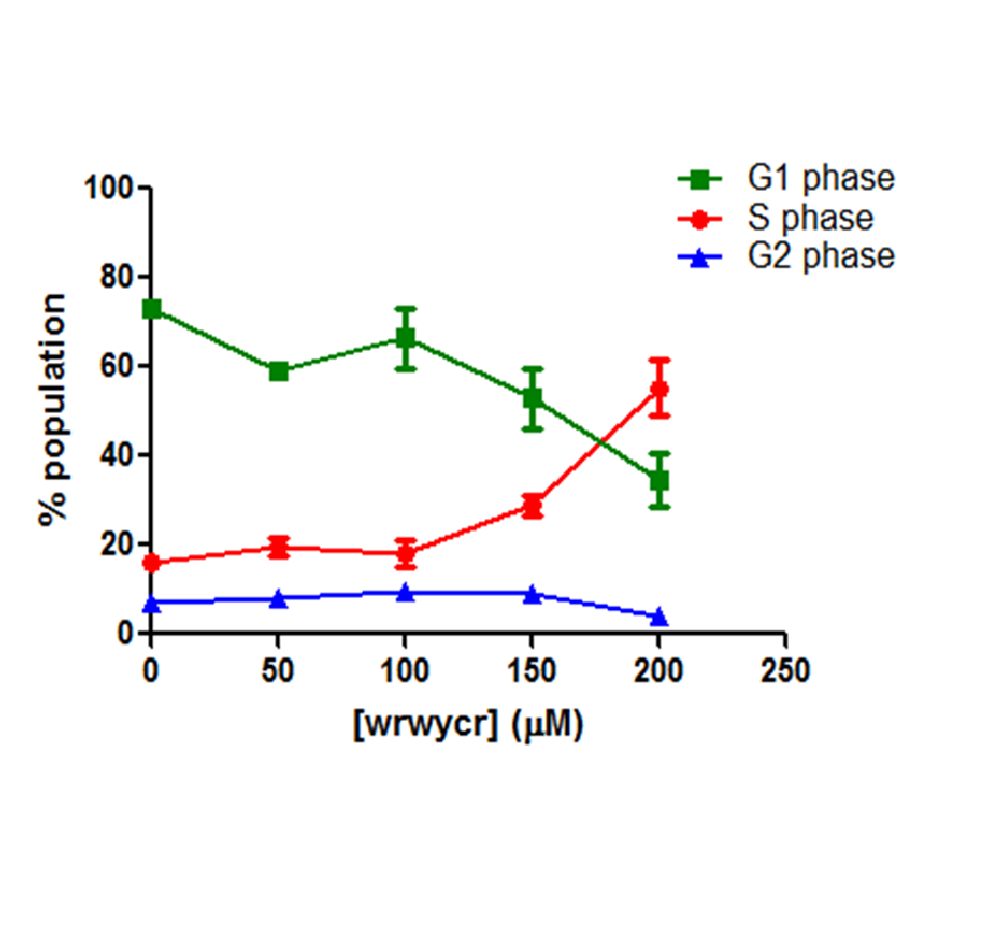

Supplement: Figure S1 — Interference with the PC3 cell cycle by wrwycr treatment for 72 h. Graphical representation of PC3 cells treated with wrwycr for 72 h from cell cycle analysis performed as the analysis shown in Figure 8B. A significant increase in the fraction of S-phase cells was observed after treatment with 200 µM wrwycr compared to treatment with DMSO, as found by one-way Anova using Bonferroni post-test analysis. (TIF) [file pone.0078751.s001.tif]

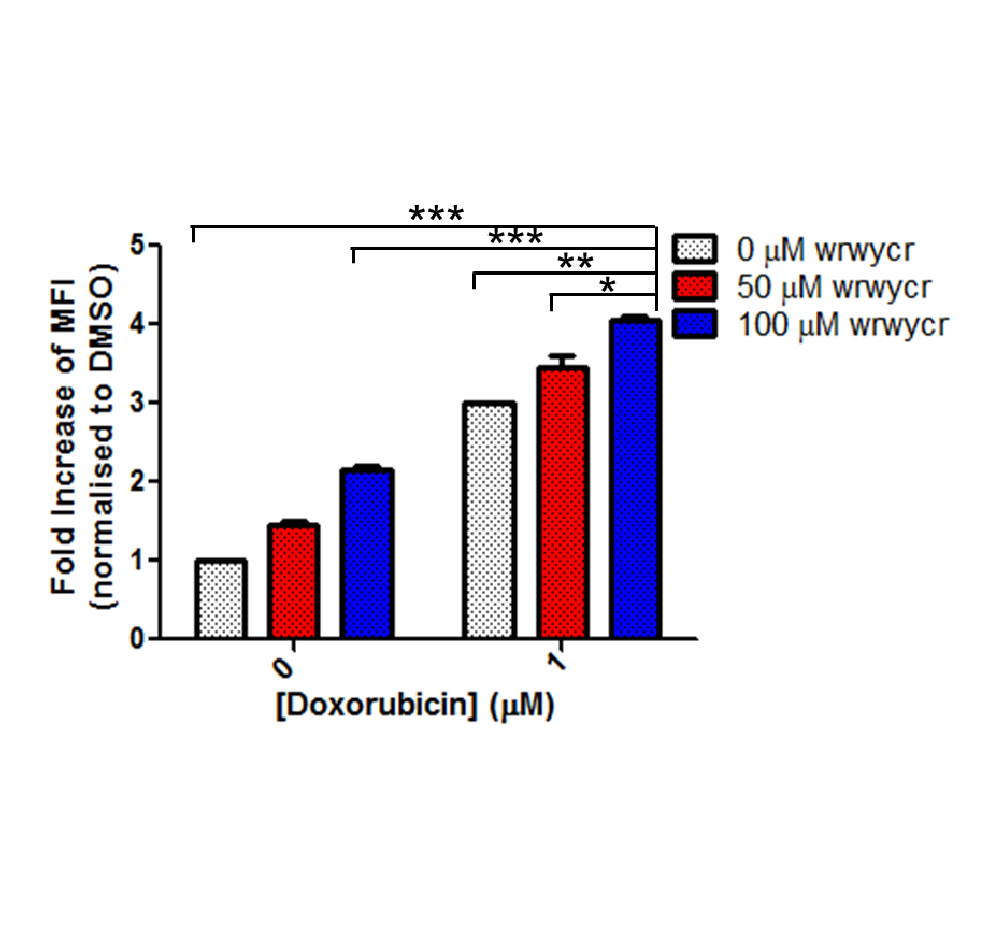

Supplement: Figure S2 — Mean Fluorescence intensity of pChk2 population increases with combination treatment of doxorubicin and wrwycr. PC3 cells were treated with 50, 100 µM wrwycr and/or 1 µM doxorubicin for 48 h and analyzed for the activation of Chk2, as described in Materials and Methods. Significance was determined with a one-way Anova using Bonferroni post-test analysis. *** indicates p<0.001, ** indicates p<0.01 and * indicates p<0.05. (TIF) [file pone.0078751.s002.tif]
